# 

**Published:** 1897-02

**Authors:** 


### INDEX TO VOLUME XIII.

ORIGINAL COMMUNICATIONS—

Abdominal Ptosis, Remarks on, Viscero-ptosis, Enteroptosis or Glenard's Disease (Taylor)........ 177
Abortion, Treatment of (Long)........ 386
Abstract of Cases Reported at Tri-State Medical Society (Kime)........ 600
Addison's Disease, Report of a Case of (Hall)........ 90
Anesthesia in Surgery, History and Introduction of (Park)........ 649
Antipyretics, Coaltar (Martin)........ 331
Artificial Local Anemia in Surgery (Esmarch)........ 601
Broken Bone-Shafts (Manley)........ 521
Bromides, Some Unexpected Effects of (Hodges)........ 529
Cancer of the Pregnant Uterus, One Hundred and Sixty-six Cases of, Occuring Since 1886 (Noble)........297 375
Cerebral Palsies in Children (Pinckney)........ 799
Cesarean Operation Under Difficulties (Allred)........ 452
Chancroid, Nitric Acid and Gum Camphor in Treatment of (Lofton) 528
Chloroform, The Action and Administration of (Grandy)........ 223
Dysentery, The Surgical Treatment of (Patterson)........ 23
Ear Affections, Remarks Pertaining to Diagnosis and Treatment of (Hoover)........ 597
Electricity in Gynecology at Howard Hospital, Philadelphia, (Massey)........ 524
Endemic Fever of the Eastern Shore of Virginia, The (LeCato)........ 14
Ferratin and Lactophenin, Remarks on (Hubbard)........ 677
Functional and Organic Nervous Diseases, Value of Certain Therapeutics in (Pope)........ 741
Galvano-Cautery in Pterygium Operations (Hobbs)........ 433
Hand Injuries, Treatment of (Nicolson)........ 366
Hypnosis, On the Definition of (Hirsch)........ 232
H. V. M. Miller, M. D., Sketch of Life and Character of (Alexander). 289
Iritis, A Peculiar Form of, Characteristic by a Gelatinous Mass in Anterior Chamber (Phillips)........ 82
Modern Medicine, Evolution of (Allen)........ 577
Morphinism, Treatment of (Stockard)........ 444
Mucous Membrane of the Nose, Throat and Ears of Infants (Rumbold)........ 145
Mucous Membrane of the Nose, Throat and Ears of Children, Practical pathology of (Rumbold)........ 217
Mucous Membrane of the Nose, Throat and Ears of Youths, Practical Pathology of (Rumbold)........ 315
Nasal Inflammation, Supplementary Treatment (Rumbold)........ 516

ORIGINAL COMMUNICATIONS—*continued.*
Otology, A Brief History of, From Its Infancy to the Present Time, (Mitchell) ..... 73
Placenta Previa, and Report of Cases (Shannon) ..... 237
Pneumonia, (Olmsted) ..... 1
Pneumonia, The Treatment of (Baird) ..... 9
Post-Typhoid Neuritis (Preston) ..... 672
Puerperal Convulsions, Prevention of (Upshur) ..... 810
Pulmonary Consumption, Remarks on (Kendrick) ..... 806
Rectum and Anus, Diseases of (Sims) ..... 664
Reminiscences of the War, Personal and Surgical (Gaston) ..... 161
Removal of Six Inches of Small Intestine, Union by Murphy Button (Cooper) ..... 729
Retrodeviations of Uterus, The Surgical Treatment of (Goelet) ..... 88
Rheumatism and Throat Diseases (Newcomb) ..... 793
Skin Disease or Blood Disease (Hutchins) ..... 361
Skin Diseases, Modern Methods in the Treatment of (Wolff) ..... 171
Sprained Ankle, Treatment of (Elkin) ..... 664
Sputum, Examination of (Johnston) ..... 815
Strangulated Hernia (Harrison) ..... 446
Syphilis as Cause of Abortion (Ouimet) ..... 733
Syphilis, Notes on the History of (Van Goidtsnoven) ..... 92
Systematic Inspection of the Eye (Stafford) ..... 674
Tonsils, On Bony Growths Invading the (Stirling) ..... 328
Tracheotomy for Membranous Croup, the After-treatment of, Observation on Three Successful Out of Four Operations, (Harbin) ..... 154
Tuberculosis, Sanitary Regulations and Prevention (Collins) ..... 511
Typhoid Fever, Epidemic (Gordon) ..... 454
Typhoid Fever, Pathological Study of (Blackford) ..... 721
Typhoid Fever, Useful Medication in (Slack) ..... 595
Urethral Strictures, Non-Operation Treatment (Valentine) ..... 505
Urethrotomy, Remarks on Technique (Goldsmith) ..... 437
Urinary Infiltration of Scrotum Mistaken for Orchitis, a Case of, Perineal Urethrotomy, Consecutive Gangrene and Death, (Manley) 80

SOCIETY REPORTS—
American Medical Association ..... 239
Atlanta Society of Medicine ..... 681, 27 335
Clinical Society of Maryland ..... 94 385
College of Physicians (Philadelphia) ..... 547 820
Medical Association of Georgia ..... 182
Richmond Academy of Medicine and Surgery ..... 460, 537 399
Southern Surgical and Gynecological Association ..... 745
Tri-State Medical Society, Alabama, Georgia and Tennessee ..... 687

CORRESPONDENCE—
A Double Monstrosity (Johnson) ..... 263
Board of Examiners and Atlanta Clinic (Anthony) ..... 695

CORRESPONDENCE—*continued.*

Early Pregnancy, A Case of (Mitchell). ........ 105
Gangrene Resulting From Bite of "Blue Gum" Negro (Ogbourn)...... 103
God and the Doctor we Alike Adore (Duffield)........ 827
In Memoriam, Dr. Robt. Battey........ 42
In Memoriam, Dr. Wm. S. Armstrong........ 43
Medical Association of Georgia (Noble)........ 826
New York Letters ........468, 553, 510, 401, 406 36
Puerperal Convulsions, My First Case, (Cross)........ 698
Tetanus and Hydrophobia (Hillebrecht)........ 614
The Fitzgerald Colony (Coe)........ 102
Who Owns the Medical Paper? (Gould)........ 761

EDITORIALS—

American Academy of Medicine, The........ 109
American Academy of Medicine and the American Medical Association, The........ 264
American Medical Association, The........195 106
Antitoxin ........ 416
College Commencements........ 113
Deaf Mutes........ 415
Disposal of the Dead........ 615
Dr. Jos. E. Allen's Address........ 833
Dr. William S. Armstrong........ 48
Drinking-Water........473 557
Epileptics, Colonization of........ 831
Famine and Plague in India........ 832
Georgia Medical Poet........ 66
Jenner........ 347
Medical Officer of Health for Georgia........ 559
Medical Degree for Thirty-five Dollars........ 702
Medicine in 1896........ 828
Negro of the South, Effect of Freedom Upon the Mental and Physical Health of........ 763
Oculist or Optician........ 562
Our Decreasing Birth-Rate........ 479
Prevention of Cholera........ 475
Roentgen's Discovery........ 52
Sanitary Clothing........ 476
Semi-Centennial of the Discovery of Anesthesia........ 701
Specialization in Medicine ........ 617
Subscription Bills and an Explanation........ 618
Subscription Price of Medical Journals and the Sample Copy Evil .. 563
Substitution in Prescriptions........ 418
The Association at Augusta........ 197
The Case of Kitson vs. Playfair........ 198
The Examining Board and Its Critics........ 703
The Medical Association of Georgia........ 112

EDITORIALS—*continued.*

To New Subscribers........................................................ 418

Tsetse Fly, The.............................................................. 413

Volume Thirteen.............................................................. 47

What's This ? Another Consumption Cure ?.............................. 50

SELECTIONS AND ABSTRACTS—

Albuminuria, Cyclic, 351; American Dermatological Association, 420; Amygdalitis, Gargle of, 782; Anal Pruritus, Treatment of, 744; Anesthesia Statistics 274; Antipyrin, Incompatible, 499; Appendix and Foreign Bodies 814; Apenta and other Waters, 624; A Physician and Surgeon's Right to Compensation, 200; Appendicitis, 277, 238; Appendix and Grape-seeds, 782; Autumnal Fevers in Southern States, 835; Baby, How to Feed, 630; Baldness, Local Treatment of, 567; Bronchitis, Acute, in Children, 278; Cancer of the Breast, 276; Cancer, The Increase of, 131; Catgut, 827; Cerebral Surgery, Present Status of, 203; Celiotomy, The After-Treatment of, 116; Chancroid, Dressing for, 482; Chancroid Extensive, Clinic Case, 131; Chrysarobin and Chrysophanic Acid, 481; Circumstances Under Which Chloroform is Preferable to Ether, 275; Cocaine, About, 488; Code of Medical Ethics, 715; Colleges, Too Many, 568; Colles's Law in a Professional Nurse, 130; Concentrated Milk, 472; Consumption in Negroes, 631: Consumptive Patients, Precautions for, 532; Corns, 567; Cutaneous Manifestations of Diabetes, 852; Diet in Skin Diseases 844; Diphtheria Antitoxin from the Clinician's Standpoint, 126; Diphtheria, Modern Treatment in Private Practice, 786; Diseases of the Skin, Sleep in its Relations to, 129; Discovery of Anesthesia, 704; Effects of Lactation on Menstruation and Impregnation, 364; Electrolysis in Dermatology, The Value, etc., 419; Endometritis and Vaginitis, 711; Erysipelas Toxins, 566; Ether and Chloroform, The action of on the Kidney, 275; Expectorants and Cough Mixture 785; Experiments with Syphilitic Serum, 841; Fever Blisters, 567; Fistula in Ano, 412; Fractures, Recent Progress in Treatment, 485; Formalin for Ringworm, 854; Gastralgia, Arsenic for, 712; Gastritis, Chronic, of Long Standing, etc., 424; Glycerin in Stomach Affections 700; Gonorrheal Patients and Marriage, 472; Gonorrhea, 126; Guiacum in Gout and Rheumatism, 422; Guaiacol, Uses of 635; Hair, Treatment of Falling, 714; Hemorrhage, Gastric, Intestinal and Rectal, 133; Hemophilia, Treatment of, 481; Herpes Zoster of Upper Extremity, 852; Hereditary Syphilis, Recent Progress in Knowledge of 778; Homeopathic and Eclectic Therapeutics, 35; Hydrozone in Gastric and Intestinal Disorders 771; Hydrogen Peroxide, 569; Insanity, Feigned, 121; Instruments, To Preserve from Rust, 488; Insanity, The Plea of, 281, Insect Bites, Unguents for, 787; Keeping an Eye on Cuba, 282; Lupus Vulgaris, Treatment of by Electroetysis, 787; Lupus Vulgaris in Family of Tuberculous Subject, 835; Medical Education, Legislation, etc., 54; Medical Experts, Practical Suggestions to, 124; Medical Expert Testimony, 629; Medical Paper, How to Write, 626 784; Medical Writing, Style in 119; Murphy Button, 762; Navel Cord, Dry Dressing for the, 63; Neuralgia, Local Treatment for,

SELECTIONS AND ABSTRACTS—*continued.*

762; Non-Nocere in Obstetrics, 117; Optic Neuritis Following Nasal Treatment, 500; Otitis Media, 494; Otitis Media and Suppuration Mastoiditis, Treatment of, 783; Otorrhea, Treatment of, 713; Oxygen in Nose and Ear Diseases, 854; Ozone as Antiseptic, 176; Points in Electro-Therapeutics, 846; Psoriasis Treated by Mercurial Injections, 130; Purulent Discharge From Ear, 850; Purulent Otitis Media, Hydrozone in, etc., 206; Pus in the Pelvis, 273; Pyosalpinx, 274; Quinsy, Treatment of, 773; Reations of Examining Boards, etc., 628; Ringworm of Skin. 482; Scalp, Germs in, 667; Sepsis, Puerperal, 273; Sleeplessness, 420; Simple Test for Albumen, 263; Skin Disfigurements, Treatment of, 620; Skin Therapy, Leadwater in, 130; Snake-Venom Antitoxin, 633; Sprained Ankle, The Treatment of by Adhesive Strips, 132; Stigmata of Degeneracy, Abuse of, 788; Strychnine in Pregnancy, 26; Surgical Hints, 680; Surgeon, Morals of a, 536; Swelled Testicle, Treatment, 614; Syphilis and Locomotor Ataxia, 499; Syphilis, Case of Reinfection, 481; Syphilitic Sores, Salve for, 482; Syphilis, Treatment of, 565; Syphilodermata, Treatment of, 781; Syringes, Serilization of, 715; Tetanus, Treatment of, 634; Thiol Better than Ichthyol, 480; Treatment of Non-Dipheritic Sore Throat, 484; Tubercular Diseases of the Skin, Excision and Skin-Grafting, 775; Umbilical Cord, Treatment of the Stump of the, 272; Urticaria, Calcium Chloride for, 482; Vaginal Discharges, Significance of, 114; Vitiligo, 480; Vulvo Vaginitis and Purulent Ophthalmia 857; Warty Growths of Genitals, 819; X Rays, Effect of on Skin, 786; X Ray in Surgery, 849.

MEDICAL ITEMS—63, 134, 208, 283, 354, 426, 489, 641, 716, 788, 855.

BOOK REVIEWS—

*Academy of Railway Surgeons,* Report of Meeting. J. H. Reed......... 502
*American Year-Book of Medicine and Surgery*.......... ......... ......... ... 70
*Anatomy.* By Henry Gray. ... ........................ 501 572
*An American Text-Book of Surgery.* Edited by W. W. Keen, M.D., and J. Wm. White, M. D.................. 360
*A Text-Book of Bacteriology.* By George M. Sternberg.................. 430
*Atlas of the Normal and Pathological Nervous Systems.* By Christfried Jakob.................. 140
*Autoscopy of the Larynx and the Trachea.* By Alfred Kirstein, M.D...... 863
*Borderland Studies.* By G. M. Gould.................. 645
*Color Vison and Color Blindness.* By J. E. Jennings.. ....... ..... ...... 70
*Compend of Diseases of Children.* By M. H. Hatfield.................. 648
*Compend of Gynecology.* By W. H. Wells.................. 648
*Dame Fortune Smiled*.................. 214
*Diagnostic Urinology.* By M. D. Hoge.................. 572
*Diets for Infants and Children.* By Louis Starr.................. 216
*Diet in Sickness and in Health.* By Mrs. Ernest Hart.................. 144
*Diseases of the Rectum and Anus.* By S. G. Gant, M D.................. 215
*Don'ts for Consumptives, etc*.................. 72
*Electricity in Electro-therapeutics.* E. J. Houston.................. 215

BOOK REVIEWS—*continued.*
*Essentials of Physical Diagnosis of the Thorax.* By Arthur M. Corwin, A. M., M.D. ........ 862
*Fundus Oculi.* By Adams Frost ........ 574
*Guide Medical Parisien* ........ 431
*Handbook of Medical Diagnosis.* By Jas. B. He rrick ........ 143
*How to Examine for Life Insurance.* By John M. Keating ........ 143
*Infantile Mortality During Childbirth and its Prevention.* By A. Brothers, ........ 288
*International Medical Annual* ........ 288 864
*Manual of Anatomy.* By Irving S. Haynes ........ 359
*Manual of Clinical Diagnosis.* By Chas. E. Simon ........ 572
*Manual of Medical Jurisprudence and Toxicology.* By Henry C. Chapman ........ 144
*Manual of Venereal Diseases.* By J. R. Hayden. ........ 573
*Medical Jurisprudence, Forensie Medicine and Toxicology.* By R. A. Witthaus and T. C. Becker ........ 142
*Miskel* ........ 141
*Moody's Magazine of Medicine.* ........ 502
*Non-Heredity of Inebriety.* By L. E. Keeley ........ 720
*Over the Hookah.* By G. F. Lydston ........ 718
*Pregnancy, Labor and the Puerperal State.* By Grandin and Jarman ........ 141
*Ptomains, Leucomains, Toxins, etc.* By Vaughan and Novy ........ 574
*Reference Book of Practical Therapeutics.* By Frank P. Foster ........ 792
*Reference Handbook of Skin Diseases.* By G. T. Jackson ........ 573
*Sickness and in Health, (In).* By J. W. Roosevelt ........ 503
*Spectacles and Eye-Glasses.* By R. J. Phillips ........ 576
*Students' Medical Dictionary.* By G. M. Gould ........ 647
*Syphilis in the Middle Ages and in Modern Times.* By Dr. F. Buret ........ 139
*System of Surgery.* By F. S. Dennis and Jno. S. Billings ........ 502
*Text-Book of Materia Medica, Therapeutics and Pharmacology.* By G. F. Butler ........ 720
*Text-Book Upon the Pathogenic Bacteria.* By Jos. McFarland, M.D.... 214
*The Functional Examination of the Eye.* By J. H. Claiborne ........ 138
*The History of Anesthesia.* By W. R. Hayden ........ 71
*The Journal of Cutaneous and Genito-Urinary Diseases* ........ 963
*The Pathology and Surgical Treatment of Tumors.* By Nicholas Senn... 67
*The Practice of Medicine.* By James Tyson, M.D ........ 862
*The Principles of Theoretical Chemistery.* By Ira Remsen ........ 861
*The Multum in Parvo Reference and Dosebook.* By C. Henri Leonard, 432
*The Three Ethical Codes.* ........ 360
*Transactions American Dermatological Association* ........ 288
*Transactions Southern Surgical and Gynecological Association* ........ 432
*Treatise on Appendicitis.* By Jno. B. Deaver ........ 646
*Treatise on Obstetrics.* By E. P. Davis ........ 646
*Treatise on Surgery.* By Roswell Park ........575 719
*Twentieth Century Practice, Diseases of the Respiratory Organs* ........ 69
*Twentieth Century Practice. Diseases of the Skin* ........ 287
*Voice Building and Tone Placing.* By H. H. Curtis ........ 287

### CONTRIBUTORS TO VOLUME XIII.

Alexander, Hooper, Esq. ........ Atlanta, Ga.
Allen, Jos. Eve, M. D. ........ Augusta, Ga.
Allred, I. P., M.D ........ Genoa, Fla.
Baird, J. B., M. D. ........ Atlanta, Ga.
Blackford, C. M., M. D. ........ Atlanta, Ga.
Collins, K. R., M. D. ........ Atlanta, Ga.
Cooper, H. P., M. D. ........ Atlanta, Ga.
Elkin, W. S., M. D. ........ Atlanta, Ga.
Gaston, J. McF., M. D. ........ Atlanta, Ga.
Goelet, Augustin H., M. D. ........ New York, N. Y.
Goldsmith, W. S., M. D. ........ Atlanta, Ga.
Gordon, W. S., M. D. ........ Richmond, Va.
Grandy, L. B., M. D. ........ Atlanta, Ga.
Hall, J. N., M. D. ........ Denver, Col.
Harbin, R. M., M. D. ........ Rome, Ga.
Harrison, V. W., M. D. ........ Richmond, Va.
Hirsch, Max, M. D. ........ Berlin, Germany.
Hobbs, A. G., M. D. ........ Atlanta, Ga.
Hodges, J. A., M. D. ........ Richmond, Va.
Hoover, F. P., M. D ........ New York, N. Y.
Hubbard, T. V., M. D. ........ Atlanta, Ga.
Hutchins, M. B., M. D. ........ Atlanta, Ga.
Johnston, J. I., M. D. ........ Pittsburg, Pa.
Kendrick, W. S., M. D. ........ Atlanta. Ga.
Kime, R. R., M. D ........ Atlanta, Ga.
LeCato, Geo. W., M. D. ........ Wachaprague, Va.
Lofton, Lucien, M. D. ........ Atlanta, Ga.
Long, J. W., M. D. ........ Richmond, Va.
Manley, Thos. H., M. D. ........ New York, N. Y.
Martin, J. A., M. D. ........ Indianapolis, Ind.
Massey, G. B., M. D. ........ Philadelphia, Pa.
Mitchell, T. E., M. D. ........ Columbus, Ga.
Newcomb, J. E., M. D. ........ New York, N. Y.
Nicolson, Wm. Perrin, M. D. ........ Atlanta, Ga.
Noble, Geo. H., M. D. ........ Atlanta, Ga.
Olmsted, J. C., M. D. ........ Atlanta, Ga.
Ouimet, J. A., M. D. ........ Montreal, Canada.
Park, Roswell, M. D. ........ Buffalo, N. Y.
Patterson, E. L., M. D. ........ Barnwell, S. C.
Phillips, S. Latimer, M. D. ........ Savannah, Ga.
Pinckney, S. G. C., M. D. ........ Atlanta, Ga.

CONTRIBUTORS—*continued.*
Pope, Curran, M. D........................................................Louisville, Ky.
Preston, Geo. J., M. D....................................................Baltimore, Md.
Rumbold, T. F., M. D......................................................St. Louis, Mo.
Shannon, Jno. R., M. D.................................................... Cabaniss, Ga.
Sims, Geo. K., M. D.......................................................Richmond, Va.
Slack, H. R., M. D........................................................LaGrange, Ga.
Stafford, H. E., M. D.....................................................New York, N. Y.
Stirling, Alex. W., M. D.................................................. Atlanta, Ga.
Stockard, C. C., M. D..................................................... Atlanta, Ga.
Taylor, Hugh M., M. D.....................................................Richmond, Va.
Upshur, J. N., M. D.......................................................Richmond, Va.
Van Goidtsnoven, E., M. D.................................................Atlanta, Ga.
Valentine, F. C., M. D....................................................New York, N. Y.
Von Esmarch, F............................................................ Germany.
Wolff, Bernard, M. D...................................................... Atlanta, Ga

